# Synthetic osteogenic extracellular matrix formed by coated silicon dioxide nanosprings

**DOI:** 10.1186/1477-3155-10-6

**Published:** 2012-01-27

**Authors:** Jamie L Hass, Erin M Garrison, Sarah A Wicher, Ben Knapp, Nathan Bridges, DN Mcllroy, Gustavo Arrizabalaga

**Affiliations:** 1 Department of Physics, University of Idaho, Moscow, Idaho, 83844, USA; 2 Department of Biological Sciences, University of Idaho, Life Sciences South Room 142, Moscow, ID 83844, USA

**Keywords:** nanosprings, nanomaterials, osteoblasts, osseointegration, calcification, bone regeneration

## Abstract

**Background:**

The design of biomimetic materials that parallel the morphology and biology of extracellular matrixes is key to the ability to grow functional tissues *in vitro *and to enhance the integration of biomaterial implants into existing tissues *in vivo*. Special attention has been put into mimicking the nanostructures of the extracellular matrix of bone, as there is a need to find biomaterials that can enhance the bonding between orthopedic devices and this tissue.

**Methods:**

We have tested the ability of normal human osteoblasts to propagate and differentiate on silicon dioxide nanosprings, which can be easily grown on practically any surface. In addition, we tested different metals and metal alloys as coats for the nanosprings in tissue culture experiments with bone cells.

**Results:**

Normal human osteoblasts grown on coated nanosprings exhibited an enhanced rate of propagation, differentiation into bone forming cells and mineralization. While osteoblasts did not attach effectively to bare nanowires grown on glass, these cells propagated successfully on nanosprings coated with titanium oxide and gold. We observed a 270 fold increase in the division rate of osteoblasts when grow on titanium/gold coated nanosprings. This effect was shown to be dependent on the nanosprings, as the coating by themselves did not alter the growth rate of osteoblast. We also observed that titanium/zinc/gold coated nanosprings increased the levels of osteoblast production of alkaline phosphatase seven folds. This result indicates that osteoblasts grown on this metal alloy coated nanosprings are differentiating to mature bone making cells. Consistent with this hypothesis, we showed that osteoblasts grown on the same metal alloy coated nanosprings have an enhanced ability to deposit calcium salt.

**Conclusion:**

We have established that metal/metal alloy coated silicon dioxide nanosprings can be used as a biomimetic material paralleling the morphology and biology of osteogenic extracellular matrix. The coated nanosprings enhance normal human osteoblasts cellular behaviors needed for improving osseointegration of orthopedic materials. Thus, metal-coated nanosprings represent a novel biomaterial that could be exploited for improving success rates of orthopedic implant procedures.

## Background

The assembly of individual cells into functional, healthy, tissue relies on the structural and morphological integrity of the extracellular matrix (ECM) [1]. Consequently, many efforts to bioengineer the ECM by mimicking its three dimensional structure at the nanoscale have been undertaken [2,3]. The goal of for these artificial matrixes is to serve as scaffolds upon which tissue development, both *in vitro *and *in vivo*, can be promoted and accelerated. Indeed, several nanomaterials, form structural complexes that efficiently allow proliferation and differentiation of various types of cells, including cardiomyocytes, epithelial cells, hepatocytes and osteoblasts [2].

Special attention has been put into making a prototypical system mimicking the nanostructures of the ECM of bone, as there is a need to find materials that can enhance the bonding between orthopedic devices and this tissue [4,5]. The most common cause of orthopedic implant failure is a suboptimal bone-implant interface, caused by the lack of integration of the implant into the bone structure (i.e. osseointegration), which leads to aseptic loosening [6]. Consequently, efforts to bioengineer bone ECM by mimicking its three dimensional structure at the nanoscale has been undertaken [2].

The process of osseointegration involves the bone healing around the orthopedic device. Healing occurs at a nanoscale level as specialized cells, osteoblasts, secrete both the organic and inorganic components of the bone ECM. The efficiency of this process relates to the degree of mineralization that occurs during the healing process [7]. A crystalline organic phase (collagen) makes up 90% of the organic component of the bone matrix while the inorganic component of bone ECM is mainly a crystalline mineral phase, hydroxyapatite crystal (HA), which makes up 65% of the bone matrix and provides the strength property of bone [8]. Besides providing a structural scaffold, the ECM is known to regulate important cellular functions such as proliferation, migration, adhesion and differentiation [2]. Thus, the ideal coating for orthopedic implants is one that mimics the morphology of the natural bone ECM and also contains chemical properties that induce osteoblast growth and development.

A novel approach to the generation of new implant materials takes advantage of the advent of nanomaterials. Foremost, the allure of nanomaterials is the significant increase in surface area they provide. By simply increasing the surface area, the nanomaterials can provide additional points on an implant for the bone to contact, which in turn improve the chances for osseointegration. Osteoblast attachment, growth, proliferation and differentiation have shown to be enhanced by various nanosurfaces. Tantala nanotubes, titanium dioxide (TiO_2_) nanotubes, carbon nanotubes and hydroxyapatite nanocrystallites have all been investigated for osteogenic properties [2,5,9].

We have recently developed a new type of nanomaterial, silicon dioxide nanosprings (NS) [10], which showed characteristics that make it a good candidate for an artificial bone ECM and thus as an implant coating. NS are a helical form of silicon dioxide nanowires that form an amorphic mat, where the thickness and density of the mat can be controlled to produce varying degrees of porosity [11]. NS form via vapor-liquid-solid procedure and can be easily grown on a variety of substrates, including metals, ceramic, and even some high temperature plastics [12,13].

**Figure 1:**
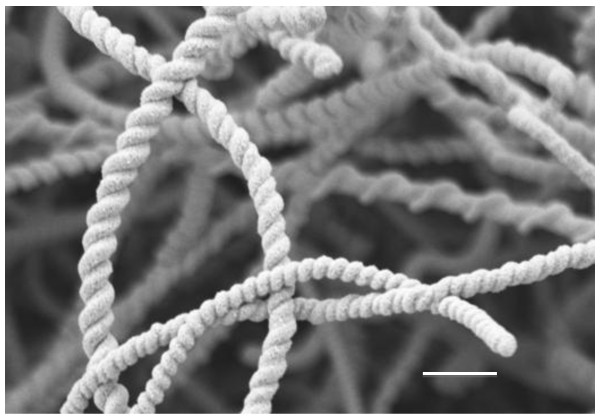
**Scanning electron microscopy of zinc oxide coated nanosprings**. Scale bar = 1 μm.

The NS are currently being explored for use in nanoelectronics, nanomechanics, and nanoelectromechanical systems [12,13]. An important feature of NS is that their surface can be enhanced with metals and metal alloy coatings (Figure 1) [13,14], and can easily be conjugated to proteins or bioactive molecules [13,14]. The helical structure of NS provides a significant increase in surface area and the potential for interaction between conjugated factors. Thus, in many regards, NS mats are an ideal matrix for cells, such as osteoblasts, upon which to grow and develop.

The nanosprings are remarkably similar to bone's collagen fiber type 1 (CF1), the main organic component of bone ECM. The NS are comparable in both size and shape to this protein [8,10]. The diameter of the average nanospring (191.8 ± 43.6 nm) is comparable to the diameter of extracellular CF1 (191 nm) [15-17]. Because CF1 is a helical crystal of repeating functional sites, of which there are many hydroxyl sites, it provides numerous bonding and attachment sites over its entire length [8]. The NS mimic this behavior because they are a helical structure, which possess uniform repeating chemical makeup of hydroxyls that provide interaction points for the osteoblasts. The addition of coatings provides the surface properties not unlike those of the hexagonal lattice formed by HA, which is bonded to CF1 [8]. Therefore, the coated nanosprings have the potential to mimic the organic and inorganic components of bone ECM and serve as an artificial surface for bone cell growth.

Here we show that NS coated with a variety of chemicals not only allow growth of human osteoblast in tissue culture, but also increase the rate at which they divide. Furthermore, we have discovered that osteoblasts grown on coated NS have a higher rate of differentiating into mature bone cells, which mineralize and potentially form mature bone. These results indicate that NS could be a useful substrate to induce osseointegration of orthopedic implants, which would reduce implant failure.

## Results and discussion

### Nanosprings can be grown on glass discs and autoclaved

To analyze the compatibility of NS with bone cell growth and development, we established a system that allowed us to easily monitor and analyze cell proliferation and differentiation. Glass cover slips are an ideal material for cell culture as they allow cell adhesion, growth of most cell types and they mount on slides for microscopy analysis. NS grown to our specifications on the glass discs using a vapor-liquid-solid method [10,11] were purchased from GoNano Inc. (Moscow, Idaho). Visual analysis of the discs after nanospring growth was performed using field emission scanning electron microscopy (FESEM) and allowed us quality control that the silica NS grown on glass formed helical structures characteristic of this nanomaterial. The nanospring density on the cover slips ranged from 0.71 to 1.01 mg/cm^2^.

Given the relative lack of information available on the interaction between osteoblasts and metal/metal alloy nanosurfaces, we also coated the NS with either of 2 different biocompatible materials, gold nanoparticles (Au), titania (TiO_2_) or with combinations of them with, or without, zinc oxide (ZnO) (i.e. TiO_2_/Au, TiO_2_/ZnO and TiO_2_/Au/ZnO). Coatings with ZnO or ZnO/Au were not done because of the sensitivity and toxicity of bone cells to zinc oxide. Toxicity of bone cells to ZnO is reported to occur at a blood level of 0.1 μg/cm^3 ^and the density of this coating on the NS, which is the actual surface contact amount, is ~ 3000.0 μg/cm^2 ^[18,19]. Even with the gold nanoparticles, which covered only 16% of the surface at most (EDS data), the ZnO level would be too high. Nonetheless, we wanted to include ZnO in some samples to examine the response of osteoblasts to the properties of conductivity, piezoelectricity and low thermal expansion that the underlayer of ZnO might provide. For this purpose we first added ZnO to the cover slips and then covered them with the TiO_2 _layer, which is likely to protect the cells against the effects of the ZnO layer.

Coating densities were calculated to be 0.5 ± 0.05 mg/cm^2 ^for titania, 3.0 ± 0.05 mg/cm^2 ^for zinc oxide and 0.015 ± 0.003 mg/cm^2 ^for gold nanoparticles. An important requirement for the utilization of nanomaterials for any biomedical purpose is the ability to sterilize the material prior to use as to avoid introduction of microbial contamination. Thus, we tested the ability of the NS grown on glass to withstand sterilization temperatures (134°C) and pressures (4 PSI or 207 Torr) for 35 minutes in an autoclave. Attachment of the NS to the glass cover slips was maintained through the autoclaving process and FESEM analysis showed no effect on the integrity or structure of the NS. In addition to FESEM, EDS was also preformed, which showed statistically (p-value < 0.0001, Anova) no change in the atomic content at the surface when evaluated at 15 and 20 kV at magnifications of 50× and 500×. The bare NS and coated NS are fabricated out of robust biocompatible metals, for which the melting temperatures are very high, lowest melting temperature for the materials used is for silicon dioxide and gold, ~1600°C and these metal/metal alloys have been widely reported for their ability to withstand sterilization procedures. Thus, NS are ideally suited for use in both *in vitro *and *in vivo *tissue studies as the can be easily sterilized using standard methods.

### Osteoblasts exhibit an enhanced proliferation rate when grown on coated nanosprings

**Figure 2:**
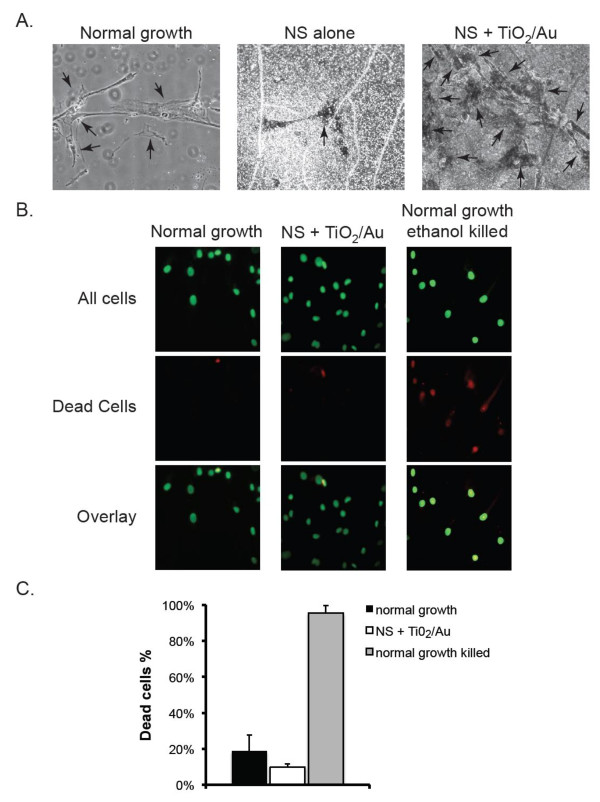
**Growth of normal human osteoblasts on nanosprings**. A. Osteoblasts were grown for 3 days on plain glass (normal growth), NS or on NS coated with titania (TiO_2_) and gold nanoparticles (Au) and imaged by contrast light microscopy. Arrows point at individual cells. B. Cells grown on normal growth conditions or on NS + TiO_2_/Au surfaces for 5 days were stained with Vybrant Green (green), which labels the nuclei of all cells and with propidium iodide (red), which preferentially stains dead cells. The overlap of both stains is shown in the last row. As a positive control for death, cells grown on normal conditions were treated with ethanol (bottom row). C. The percentage of cells dead cells for the three types of cultures in B was determined for a total of 500 cells by dividing the number of propidium iodide positive cells by the number of Vybrant Green positive cells. Data were compiled from 3 independent experiments. Error bars represent standard deviation.

To test the effect of the NS on osteoblast proliferation, 10^4 ^normal human osteoblasts (NHOs) were seeded on plain cover slips, cover slips with bare NS or cover slips with the different coated NS. At the end of the 5-day incubation of NHOs on the different discs it was visually apparent that the number of cells were significantly (p-values < 0.0001, Anova) greater on the cover slips containing NS coated with either TiO_2_/Au, or TiO_2_/Au/ZnO, when compared to either plain cover slips (Figure 2A). We tested whether the observed cells were alive by staining them with Vybrant Green, which detects all cells gives and with propidium iodide (PI), which preferentially stains dead cells (Figure 2B). Quantification of the staining experiment, showed similar levels of cell death in both normal growth and TiO2/Au coated NS (19 ± 9% and 10 ± 2% respectively). As a positive control, we treated cells grown on normal growth conditions with ethanol, which killed 96 ± 4% of the osteoblasts (Figure 2C).

**Figure 3:**
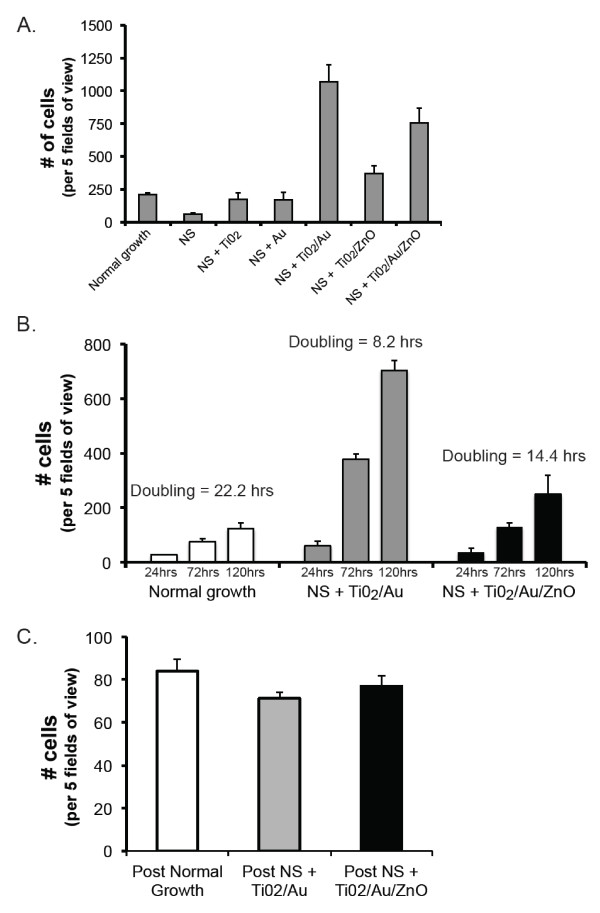
**Effect of different coated NS on cell proliferation**. A. Cells were allowed to grow for 5 days on either plain glass (normal growth), plain nanosprings (NS) or NS coated with gold nanoparticles (Au), titania (TiO_2_), titania and gold (TiO_2_/Au), titania and zinc oxide (TiO_2_/ZnO) or titania, gold particles and zinc oxide (TiO_2_/Au/ZnO). The total number of live cells, as determined by calcein blue staining, was counted in five fields of view for each sample. Data were compiled from 3 independent experiments. Error bars represent standard deviation. B. Total number of live cells in five fields of view was determined for cells grown for 1, 3 or 5 days on normal growth, NS + TiO_2_/Au or NS + TiO_2_/Au/ZnO. Data were compiled from 3 independent experiments. Error bars represent standard deviation. Doubling time between 1 and 5 days was determined by dividing 96 (the total number of hours between those two time points) by the ratio of number of cells at day 5 to the same at day 1. C. Five hundred cells harvested from cultures grown for four days on normal conditions, NS + TiO_2_/Au or NS + TiO_2_/Au/ZnO were propagated on normal culture conditions for 3 days. Total number of cells in five fields of view was determined for each sample. Bars represent average of 3 independent experiments. Error bars are the standard deviation.

The observation that more cells could be seen in the cultures that contained NS was confirmed by careful quantification of the number of cells on each one of the different discs. Cover slips with nanosprings coated with either TiO_2_/Au, and TiO_2_/Au/ZnO had a higher number of cells per field of view, when compared to either plain cover slips or cover slips with uncoated nanosprings (Figure 3A). NS coated with TiO_2 _and ZnO also showed an increase in the number of cells relative to normal growth conditions, albeit not as great an extent as what is observed with coating combinations that contain Au (i.e. TiO_2_/Au and TiO_2_/Au/ZnO, Figure 3A). In the cases where the coatings enhanced cell proliferation, the enhancement only occurred when in conjunction with the NS, i.e. the control coatings did not produce enhanced proliferation and only resulted in normal growth (additional file 1).

Our proliferation studies strongly suggest that osteoblast divide at a faster rate when grown on NS along with either TiO_2_/Au or TiO_2_/Au/ZnO coatings. To more precisely explore this phenomenon we added the same number of cells to plain cover slips and cover slips containing NS with those two different coatings and counted the number of live cells after 24, 72 and 120 hrs. (Figure 3B). While the doubling rate between 24 and 120 hours for cells grown on plain glass was every 22.2 hours, cells grown on NS with TiO_2_/Au and NS with TiO_2_/Au/ZnO double their numbers every 8.2 and 14.4 hours respectively, during the same time period (Figure 3B). Thus, NS coated with titania and gold nanoparticles accelerated the division of osteoblasts by almost three times of what is observed under normal growth conditions.

The pores and the scaffolding present in bone ECM are known to play a role in differentiation, likely by providing pockets of surface charges in which projections from the osteoblasts can sense the lack of mineralization [8]. Pore size in natural bone varies quite a bit (20-400 μm) depending on location and function needed such as support or a fulcrum point [1]. The nanospring mat can also be fabricated to achieve different levels of porosity and the pores on the samples used for this study ranged between 5-25 μm. Consequently, the porous nature of the NS mat might have contributed to the effect of the coated NS on osteoblasts osteogenic behavior by providing a greater number of gaps that can interact with the osteoblasts and be filled with bone matrix.

The enhanced proliferation with coated NS, confirms our hypothesis that the mats of NS, because of the similar morphology to collagen, provide an excellent material upon which to grow NHOs. Conversely, these findings also indicate that the morphological similarities between NS and collagen alone are not sufficient to enhance proliferation and that surface chemistry and stoichiometry are equally important, as borne out by the lack of enhanced proliferation on uncoated NS. To the best of our knowledge, this is the first study of nanomaterials to definitely demonstrate the equal importance of the nanomaterial's morphology and stoichiometry on osteoblast proliferation.

The bare NS used in this study were completely hydrophilic and thus we hypothesize that a monolayer of water forms around the bare NS, which in turn would block binding of proteins and cells as reported with other completely hydrophilic surfaces [20]. Thus, the decrease in proliferation on hydrophilic NS without coatings might be due to a decrease in attachment as oppose to a direct effect on the NHO proliferation ability. It was noted during incubation, that the NHO stayed rounded up instead of becoming their typical elongated columnar morphology and we did not observe a higher percent of dead cells on cultures grown on plain NS (additional file 2).

The coatings that had the biggest effect on cell proliferation where those that combined TiO2 and Au (Figure 3A). Interestingly, neither of these materials enhanced proliferation over the control when used by themselves to coat the nanosprings. Thus gold and titanium on nanosprings appear to have a synergistic or cooperative effect to enhance the doubling rate of osteoblasts. Why these materials are having such an effect is not understood at this point. One possibility is that together they modify the 3-D structure of the NS array in a way that is more conducive to osteoblast division. It is also possible that these metals are providing additional sites for the osteoblasts to attach to. It has been shown that many proteins found in bone EMC, including fibronectin and osteonectin attach to gold nanoparticles [21,22]. Thus, it is possible that the addition of gold nanoparticles imparts sites of protein attachment along the length of the nanosprings thus enhancing proliferation. Similarly titania has been shown to be bioactive and biocompatible for osteoblasts and these cells can attach to this metal alloy [5]. Previously, it has been found that titania surfaces have an alternating chemical composition between hydrogen and hydroxyls and do not form a water monolayer [23]. So the titania might be influencing osteoblast proliferation directly by providing sites of attachment or indirectly by eliminating a water shell. Consistent with either of these possibilities, osteoblasts were noted to attach more efficiently to the TiO_2 _coated nanosprings (data not shown).

### The fast propagation rate of cells grown on coated nanosprings is reversible

It is conceivable that when grown on NS, osteoblast are transformed to faster dividing cells, because they either mutated or differentiated into a different type of cell. To determine whether the fast proliferative phenotype of osteoblasts grown on NS constitutes a permanent change in the cells we tested the proliferation rate of cells previously grown on NS. For this purpose, cells grown for 4 days on either normal cell culture conditions, TiO2/Au coated NS, or TiO2/Au/ZnO coated NS were harvested and the same number of cells were added to plain cover slips to monitor their growth. Regardless of how the cells were grown previous to the test, we observed the same number of cells 4 days later. This indicates that the high proliferation rate of the cells grown on NS is a reversible trait and that the NS did not permanently transformed the cells in the time frame tested (Figure 3C).

### Coated NS enhance osteoblast development

**Figure 4:**
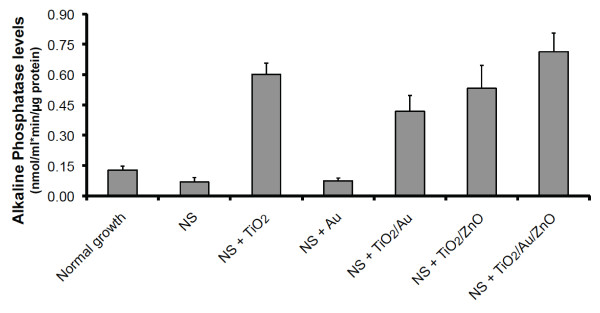
**Differentiation of osteoblasts grown on coated nanosprings**. Cells were grown for 36 days under normal growth conditions or on the NS samples indicated in the graph. Levels of alkaline phosphatase activity normalized to total amount of protein were calculated for each sample. Bars represent average of 3 independent experiments. Error bars are the standard deviation.

Osteoblasts need to differentiate into cells that produce bone matrix, collagen and many other osteogenic factors to support osseointegration. This process involves differential expression of certain proteins. One of these factors, alkaline phosphatase, can be used as biomarker to determine whether and to what level, osteoblasts have differentiated into matured bone forming cells [16,24]. Consequently we tested the levels of alkaline phosphatase activity of cells grown for 36 days on either normal conditions or NS coated with the various materials used in the proliferation studies. Since after 36 days the number of cells on each of the cover slips would be different we normalized our results to the total amount of protein in the tested sample. While cells grown on plain NS and Au coated NS showed similar levels of alkaline phosphatase as those grown on normal conditions (Figure 4), osteoblast grown on the TiO_2_, TiO_2_/Au, TiO_2_/ZnO and TiO_2_/Au/ZnO coated NS exhibited significantly (p-value < 0.0001, Anova) more alkaline phosphatase activity (4.70, 3.27, 4.16 and 5.57 times more respectively) than control cells (Figure 4). Thus, coated NS not only enhanced the rate at which osteoblast divide, but also influenced their ability to develop into mature forms of this type of cells. The control coatings also showed some variance but not to the degree of the coated NS (additional file 3).

In terms of differentiation, the presence of TiO_2 _appears to have an important osteogenic effect. The TiO_2 _thin coating is expected to be insulative and effectively hold charges. Furthermore, this anatase form of titania is expected to provide a negative charge shell in presence neutral pH cell media [25]. The conductivity of bone and the piezoelectric effects of CF1, which produces negative surface charges, are known to be instrumental in bone healing [8]. Thus, TiO_2 _may enhance differentiation by affecting the ionic environment, or providing the electrostatic charges that normally promote osteoblast responses during bone growth *in vivo*.

### Calcium salt deposition is enhanced in osteoblasts grown on coated nanosprings

**Figure 5:**
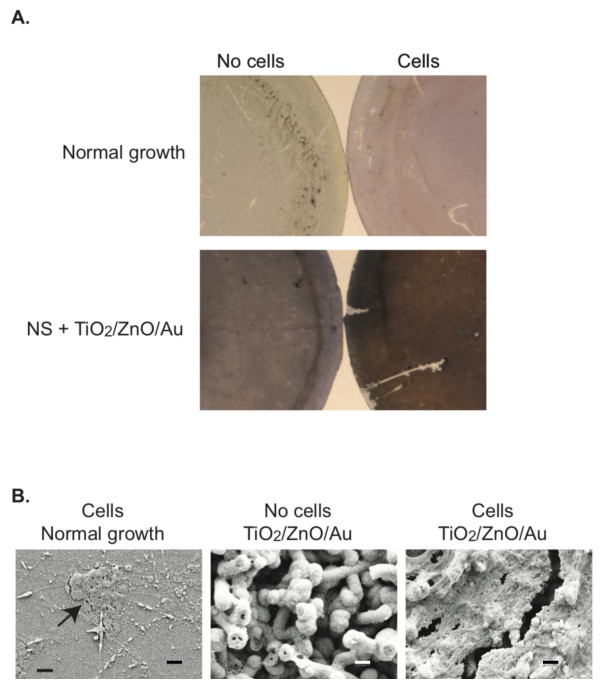
**Calcium salt deposition by cells grown on coated nanosprings**. A. Cells were grown on normal growth conditions or on NS + TiO_2_/Au/ZnO for 36 days. Cultures were stained by the Von Kossa staining method, which results in all nuclei being labeled in red, and calcium salt mineralized nodules staining brown to black depending on the level of calcification. For comparison Van Kossa staining of discs with nanosprings but no cells are included. B. Cultures shown in A were evaluated by FESEM at a magnification of 10.00 KX. Scale bar = 1 μm.

Calcium salt deposition by bone cells is one of the key cellular events needed for effective osseointegration since it leads to mineralization of the bone matrix [26]. Accordingly, we determined whether the TiO2/ZnO/Au coated NS, which was shown to be best at inducing osteoblast maturation (Figure 4), were also best at inducing calcium deposition. Calcium salt deposits were visualized using the Von Kossa staining method, in which the mineral deposits can be detected as brown to black spots depending on the level of calcification [27]. We detected a considerable number of dark spots as well as a uniform dark brown staining with cells grown for 36 days on the TiO_2_/ZnO/Au coated NS (Figure 5A). By contrast, there is minimal deposition of calcium salt nodules with the cells grown under normal conditions as indicated by the lack of brown or black spots, although many cells (red staining) are present in the sample (Figure 5A).

To quantitate the Von Kossa staining results we calculated the percentage of the area covered by black spots on randomly pick fields of view using MetaMorph™ image analysis. In normal growth conditions, we detected 0.104 ± 0.089% coverage of black stain in the areas analyzed, which was not different then what was observed with the same surface in the absence of cells. By contrast, cultures with cells grown on TiO2/ZnO/Au coated NS exhibited 60.18 ± 11.50% coverage by the stain, which represented a 11.51 fold increase over what was detected when the same surface without cells was stained. Thus, consistent with the fact that cells grown on coated NS are differentiating into bone forming cells, these cells exhibit the ability to deposit calcium salts and presumably to induce calcium deposition.

To verify the presence of bone matrix on those samples, we directly visualized them using FESEM at a magnification of 10.00 KX. While normal growth cultures showed some clusters of what appears to be hydroxyapatite crystals (Figure 5B; arrow), it contained dramatically less crystal formations than the cultures grown on NS + TiO_2_/ZnO/AuNP, which were almost completely covered uniformly in crystals (Figure 5B).

To confirm and quantify the mineralization of cultures grown on coated NS we performed energy dispersion spectroscopy (EDS) on the Van Kossa stained samples to measure the amounts of various elements. Table 1 shows the relative atomic percentages of bone primary elements for samples of cells grown on plain glass, and glass covered with NS + TiO_2_/ZnO/Au. Consistent with what we observed in the differentiation analysis, cultures grown on the coated NS promoted the highest levels of Ca and P deposition. In addition, this sample had a high percentage of silver due to the higher level of Von Kossa stain, which is the indicator for calcium salt deposition. Normal bone Ca/P ratios vary greatly in healthy bone depending on a number of variables such as anatomical location, age, sex and exercise level [28]. Kourkoumelis' study showed rat mature leg bones had a Ca to P ratio that ranged from 1.85 to 1.96. The Ca to P ration in our samples is 0.09 in early development of our best bone tissue culture sample.

**Table 1:** Percent Ca, P and Ag in relation to total% atoms on surface of cultures grown on either plain glass discs or NS coated with TiO_2_/ZnO/Au as determined by energy dispersion spectroscopy.

Element	Glass disc	NS + TiO_2_/ZnO/Au
**Ca**	0	0.17

**P**	0.11	1.80

**Ag**	0.12	2.53

**Figure 6:**
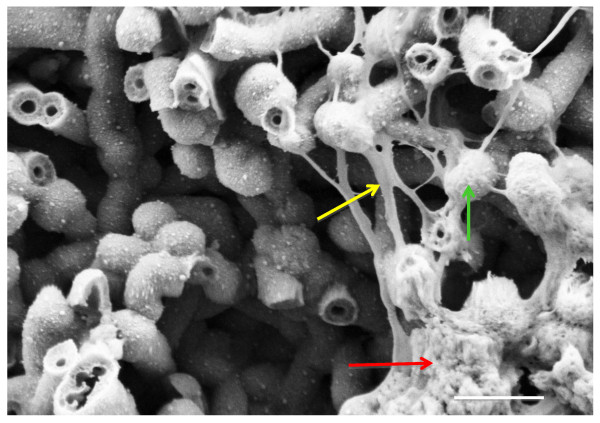
**FESEM of Bone Matrix Deposition on Coated Silicon Dioxide Nanosprings**. Hydroxyapatite crystals (red arrow) formation are seen along with filopodia (yellow arrow) from normal human osteoblasts (green arrow) on a mat of silicon dioxide nanosprings after 36 days incubation at 37°C and 5% CO. The coating on the nanosprings was titania, zinc oxide and gold nanoparticles.

Mineralization appears to be independent of the enhanced proliferation effect of NS, since we noted more mineral deposition on TiO_2 _coated nanosprings, which did not enhance proliferation (Figure 3A). There were a variety of amount of salt deposition with the different coatings on the nanosprings but as in the differentiation study, the nanosprings coated with TiO_2_/ZnO/Au proved to be the best performer. This was confirmed with FESEM where hydroxyapatite crystals along with the NHO and the filopodia could be seen (Figure 6). Thus, coated nanosprings were instrumental in bone matrix deposition and had a stimulatory effect on this activity of the osteoblast.

### Potential applications of coated nanosprings

Treatment of disorders of the musculoskeletal system, such as skeletal deformities and traumatic injuries that result in fractures, usually involve the use of orthopedic implants. One of the key challenges in this field is assuring that orthopedic implants are permanently and securely set in place. Due to the importance of the nanostructure of the bone ECM in tissue formation and development, significant efforts have been made in the engineering of biomaterials that can mimic its three dimensional characteristics of the ECM. To be of use in the in the enhancement of orthopedic implants these artificial matrixes need to serve as scaffolds for tissue growth, be easy to be grown on a variety of surfaces, able to be sterilized and have the potential for being functionalized with growth factors and proteins. In this respect, the metal-coated NS might represent an ideal nanomaterial to use as a coating on devices that need to integrate into bone.

While silica nanosprings are usually grown on silicon wafers or aluminum foil substrates for industrial uses, they can be mass-produced on a variety of surfaces including glass, titanium and ceramics. The NS grown on glass proved durable in our long-term tissue culture experiments, as we never observed delamination of the NS from the glass surface. Another advantage of the nanosprings we tested over other nanomaterials is their resistance to strong sterilization methods such as high temperature. Autoclavable materials are preferable over ones that require alcohols or ultraviolet light sterilization, since heat sterilization is easier, relatively less expensive and accessible in most medical settings.

Furthermore, the presence of metal alloys increases the versatility of this nanomaterial since the metals can serve as acceptors for the addition of proteins and other functionalizing factors. The use of AuNP as a coating, in particular, would allow us to use established surface functionalizing chemistry that can be exploited to add osteogenic factors or HA to further promote bone formation [29,30]. Potential candidates to be attached to the NS include the bone morphogenic protein-2 (BMP-2) and HA. BMP-2 is well known to stimulate bone deposition by osteoblast [31]. While BMP-2 has been added directly to the surface of orthopedic implants, it did appear to aid osseointegration, most likely because of loss of the protein from the orthopedic device's surface during implantation [6]. Introducing the protein attached to coated nanosprings may increase the adherence of the BMP-2 to the implant's surface and potentially its stability.

The other coatings we tested also proved beneficial to the propagation and development of human osteoblasts and increased the usability of NS as an implant coating. Titanium is a very common metal for many orthopedic implants because of its strength and non-corrosive ability [32]. The titania coating used in our experiments is in the anatase form, which has been shown to be more bioactive and biocompatible to human osteoblasts [33]. The other metal alloy coating, zinc oxide, which is commonly used in dental cements [34] provides interesting electrochemical properties to our nanospring coatings, and we hypothesize that this zinc oxide coating has the same crystal hexagonal structure as HA.

## Conclusions

The results presented here show that coated nanosprings can enhance the propagation, development and ability to deposit calcium salt of human osteoblasts. Furthermore, our studies allowed us to compare the responses of human osteoblasts to different metal/metal alloys being used as coating for the nanosprings, which showed unique results between proliferation and differentiation/calcium salt deposition behaviors. It is our hypothesis that the nanosprings along with their coatings provided a scaffold for controlled and guided growth as well as additional surfaces and elements for attachment. The ease of the NS to be attached to different surfaces at different densities, autoclaved for sterility and coated with a variety of metal alloys along with its unique morphology are desired traits that can be exploited to enhance osseointegration of biomaterials into living bone.

## Methods

### Cell line and culture

Normal Human Osteoblasts (NHO) supplied by Lonza Inc. (Walkersville, MD) were used for all experiments. We followed the company's instructions for preparing subcultures, calculating cell count, cell viability and seeding density. Cells were grown at 37°C and 5% CO_2 _on osteoblast growth medium (OGM), which consisted of osteoblast basal medium (Lonza, Inc.) supplemented with Clonetics SingleQuots (Lonza, Inc.) which included fetal bovine serum (FBS), ascorbic acid, and gentamicin/amphotericin B. The OGM was made following the company's specifications.

### Production of silicon dioxide nanosprings™ on glass discs

Nanospring mats were grown, to our specifications, via a vapor-liquid-solid (VLS) mechanism and purchased from GoNano Technologies, Inc. A detailed description of the process was previously reported by McIlroy et al. [10] and Wang et al. [11]. Nanosprings were fabricated on 12 mm diameter microscope cover glass discs (Fisher Scientific) in an atmospheric furnace at a constant O_2 _flow rate using a gold layer as a catalyst and a proprietary silicon precursor [US Patent application 11/993452 filed, 2010]. The growth time was approximately 15 minutes, which produced an 80-μm thick nanospring mat.

Atomic layer deposition (ALD) was used to coat the nanosprings with zinc oxide (ZnO) and titania (TiO_2_) [35,36]. ALD coatings were carried out in a tube furnace maintained at 170°C for diethyl zinc and 300°C for titanium tetrachloride (TiCl_4_) [35,37]. Between each pulse of precursor, or water, which was used as a reactant, the system was purged with N_2 _to ensure that the precursors reacted only at the surface of the growing film [38]. For gold coating, glass discs covered with nanosprings were immersed in a gold tetrachloride (AuCl_4_) solution in reagent grade ethanol. After air-drying at room temperature, the samples were reduced in an atmosphere of H_2 _and Ar for 15 minutes at 300°C. The samples were then cooled down to room temperature in Ar atmosphere to avoid oxidation.

Pre and post coating weights along with FESEM dimension of the NS were used to calculate density and determine the% weight of coating/NS for each glass disc. To verify morphology and quality of the NS mat after growth and coating procedures, all samples were analyzed visually with field emission scanning electron microscopy (FESEM).

### Cell Culture on Silicon Dioxide Nanosprings™ coated glass discs

NS coated glass discs were rinsed with deionized water and then washed for 10 minutes with 70% ethanol. The NS samples were air dried and placed in autoclave sleeves and conventionally autoclaved for 20 minutes. NS discs were place in wells of tissue culture 24 well plate and rinsed once with OGM. Fresh OGM was then added and plates were incubated at 5% CO_2 _37°C for 30 minutes before adding osteoblasts.

### Quantification of cell proliferation

10, 000 cells were added to each well containing glass cover slips. The plates were incubated at 37°C, 5% CO_2 _for 5 days. At days 1 and 3, culture media was replaced with 0.2% calcein blue (Molecular Probes, Invitrogen) in OGM and the plates were incubated for 15 minutes at 37°C with 5% CO_2_. Calcein blue stain was then rinsed twice with N'-2-hydroxyethylpiperazine-N'-Ethanesulfonic Acid (HEPES, pH 7.37, Lonza Inc.) and stained cells, which represent live cells, were counted in 5 views at 200× using a Zeiss Axiovert 40 CFL microscope. After counting, cells were washed 3 times with warm OGM and returned to tissue culture incubator to continue growth; this gives a true proliferation rate for a given culture. At day 5 of the experiment cells were terminally stained with Vybrant Green (Invitrogen) to detect all cells and propidium iodide (PI, Invitrogen) to detect dead cells. In specific, wells were rinsed twice with HEPES followed by addition of 0.06% PI, 0.16% vybrant green in PBS. After 20 min incubation at 37°C, the wells were washed with PBS two times. Cells were visualized at 200× with Zeiss Axiovert 40 CFL microscope, and the number of Vybrant Green positive and PI positive cells on 5 fields of view was counted. For each randomly selected field of view both the total and dead cells were counted before moving to the next viewing field. Images of characteristic fields of view were captured using a Canon Powershot G6 camera. There were a total of 3 testing cover slips for each coating being tested in each independent experiment and the experiment was repeated for a total of 3 independent assays. Thus, for each condition we counted cells in a total of 9 cover slips.

To determine if the NS permanently affected cell proliferation, 2, 000 osteoblasts were allowed to grow on control glass discs and coated NS discs for 4 days. After that point cells were detached and collected from the cover slips using the ReagentPac™ system (Lonza Inc.) according to manufacturer's instructions. 500 cells harvested form each of the different cultures were then added to plain glass cover slips allowing them to grow for 3 days at which time the number of live cells was determined using calcein blue stain.

### Alkaline phosphatase measurements

Control glass discs and the respective NS containing discs were placed in 24-well plates and a total of 10, 000 osteoblasts were added to each to the well. Cultures were then incubated at 37°C, 5% CO_2 _in OGM for 36 days based on published methods [16]. To maintain the pH and nutrient levels, media was changed every 4 days during this incubation period. At the end of the 36 days incubation, discs were carefully removed from the wells and placed into empty wells and then washed with HEPES two times before adding triton X-100 at 1% (500 ul/well) to lyse cells. The plates were incubated for 2 hours at 37°C, 5% CO_2 _and then lysates were transferred into microcentrifuge tubes and three rounds of freeze/thaw cycles were done at -80°C. QuantiChrom™ Alkaline Phosphatase Assay (BioAssay Systems) was used to test alkaline phosphatase levels according to manufacturer's instructions. As part of this process an EL 808 ultra microplate reader (Biotek Instruments Inc.) spectrometer was used to read emission at 405 nm. Determining total protein level for each sample normalized the readout of alkaline phosphatase activity. Total protein levels were determined using the BCA (bicinchoninic acid) protein assay (Pierce Incorporated) according to manufacturer's instructions.

### Calcium deposition studies

For calcium salt deposition experiments, osteoblast growth media was supplemented with hydrocortisone (200 nM) and β-glycerophosphate (2.0 mM). We added 10, 000 cells per sample and incubated the cultures at 37°C, 5% CO_2 _for 36 days while changing media every 3-4 days. Mineral staining was done with Von Kossa stain system (American MasterTech). In brief, media was removed and 2 mls of 3.7% formaldehyde with PBS (1:9) was added to each well and incubated for 20 minutes. All samples underwent a serial alcohol dehydration process (i.e. 70% ethanol for 10 minutes, 80% for 10 minutes, 90% for 20 minutes, and rinse with absolute alcohol). The serial dehydration process was done at this point to promote better imaging of the biologic material with the FESEM [39]. Each sample was then rinsed three times with deionized water. Staining protocol by the manufacturer was followed. The disks were viewed under light microscopy to see the dark brown or black metallic silver stain positively identifying phosphate anions of the calcium salt deposition while the cells stained red. Imaging was done using a Leica MZ16F fluorescent stereoscope, a Leica DFC420 color digital camera and Leica Application Suite software.

The % coverage of positive Von Kossa stain/area was determined by using MetaMorph™ software. Three areas of the same size on each sample were examined from the tiff image obtained from the Leica DFC420 camera. We set the color threshold to cover half of the gray scale and minimized the red hue to avoid detecting the red color contributed by gold nanoparticles, modifying a Von Kossa quantification method (BD Biosciences, technical bulletin #444). The modified method excludes less developed nodules that stained yellow to light brown. The set parameters allowed pixels to be counted that reached the resorbed part of the histogram, which represented a well-developed calcium mineral nodule. The software attained these counts that reached the set threshold in a total specified area and calculated the% threshold/area.

### Scanning Electron Microscopy and Energy Dispersion Spectroscopy

Energy dispersion spectroscopy (EDS) and FESEM was done on the samples taken from the Von Kossa calcium salt deposition study. EDS was first done using 15 kV and 20 kV, in different areas of the discs at 70 X, 300 × and 5 KX. The results of the different magnifications all had very similar quantitative and% atom content for the particular disc. The samples were coated with gold for the FESEM images (SPI-Module™ Sputter Coater; West Chester, PA) for 50 seconds. FESEM images were taken of each disc at 500×, 5 KX, 10 KX and 20 KX using Zeiss SmartSEM, with a Gemini electron gun [16].

## List of abbreviations

ECM: extracellular matrix; HA: hydroxyapatite crystal; TiO_2_: titanium dioxide; NS: silicon dioxide nanosprings; CF1: collagen fiber type 1; FESEM: field emission scanning electron microscopy; Au: gold nanoparticles; ZnO: zinc oxide; NHO: normal human osteoblasts; PI: Propidium Iodide; EDS: energy dispersion spectroscopy; BMP-2: bone morphogenic protein-2; OGM: osteoblast growth medium; FBS: fetal bovine serum; ALD: atomic layer deposition; HEPES: N'-2-hydroxyethylpiperazine-N'Ethanesulfonic Acid.

## Competing interests

A patent application was filed with the content of this article, through the University of Idaho. There is no other competing interest.

## Authors' contributions

JH, EG and SW contributed to the tissue culture procedures, bioassays and fluorescent images. JH and BK carried out the data analysis and cell counts. JH, NB and DM were responsible for quality control and FESEM images of the coated nanosprings. JH, DM and GA drafted the manuscript. GA and JH preformed the statistical analysis. All authors read and approved the final manuscript.

## Supplementary Material

Additional file 1 **Effect of different metal coatings on cell proliferation in the absence of NS**. Cells were allowed to grow for 5 days on either plain glass (normal growth), or cover slips coated with gold nanoparticles (Au), titania (TiO2), titania and gold (TiO2/Au), titania and zinc oxide (TiO2/ZnO) or titania, gold particles and zinc oxide (TiO2/Au/ZnO). The total number of live cells, as determined by Vybrant Green and PI, was counted in five fields of view for each sample. Data were compiled from 3 independent experiments. Error bars represent standard deviation. Click here for file

Additional file 2 **Viability of osteoblasts grown on nanosprings**. Cells were allowed to grow for 5 days on either plain glass (normal growth), or cover slips coated with gold nanoparticles (Au), titania (TiO2), titania and gold (TiO2/Au), titania and zinc oxide (TiO2/ZnO) or titania, gold particles and zinc oxide (TiO2/Au/ZnO). Cultures were stained with Vybrant Green (green), which labels the nuclei of all cells and with propidium iodide (red), which preferentially stains dead cells. The percentage of cells dead cells was determined for a total of 500 cells by dividing the number of propidium iodide positive cells by the number of Vybrant Green positive cells. Data were compiled from 3 independent experiments. Error bars represent standard deviation. As a positive control, we treated cells grown on normal growth conditions with ethanol, which induces death. Click here for file

Additional file 3 **Effect of metal coatings on osteoblast differentiation**. Cells were grown for 36 days under normal growth conditions or glass cover slips coated with the metals indicated in the graph. Levels of alkaline phosphatase activity normalized to total amount of protein were calculated for each sample. Bars represent average of 3 independent experiments. Error bars are the standard deviation. Click here for file
