# Supplementary figures and images for: Synthetic osteogenic extracellular matrix formed by coated silicon dioxide nanosprings
Source: J Nanobiotechnology. 2012 Jan 27;10:6. doi: 10.1186/1477-3155-10-6 (PMC3276422; doi:10.1186/1477-3155-10-6)

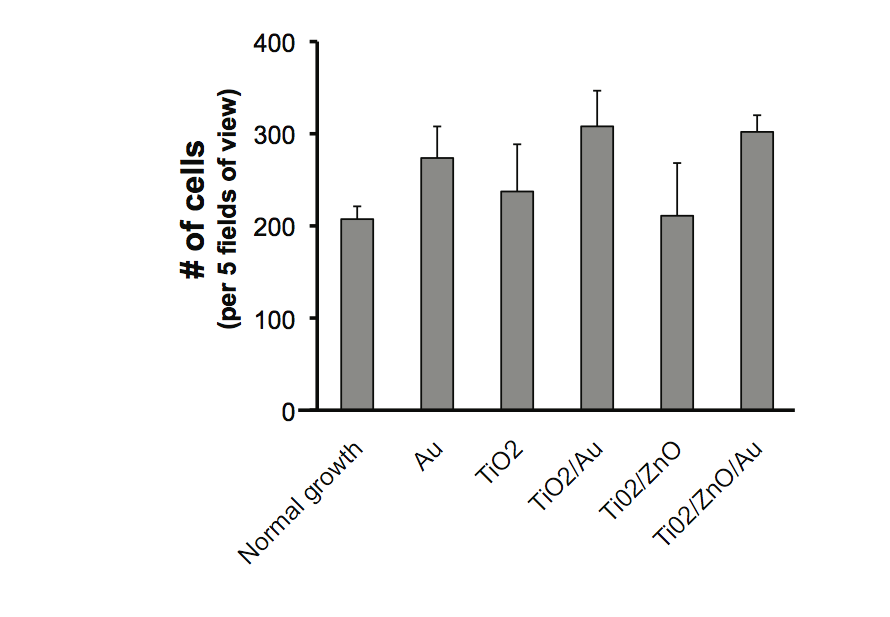

Supplement: Additional file 1 — Effect of different metal coatings on cell proliferation in the absence of NS. Cells were allowed to grow for 5 days on either plain glass (normal growth), or cover slips coated with gold nanoparticles (Au), titania (TiO2), titania and gold (TiO2/Au), titania and zinc oxide (TiO2/ZnO) or titania, gold particles and zinc oxide (TiO2/Au/ZnO). The total number of live cells, as determined by Vybrant Green and PI, was counted in five fields of view for each sample. Data were compiled from 3 independent experiments. Error bars represent standard deviation. [file 1477-3155-10-6-S1.DOC]

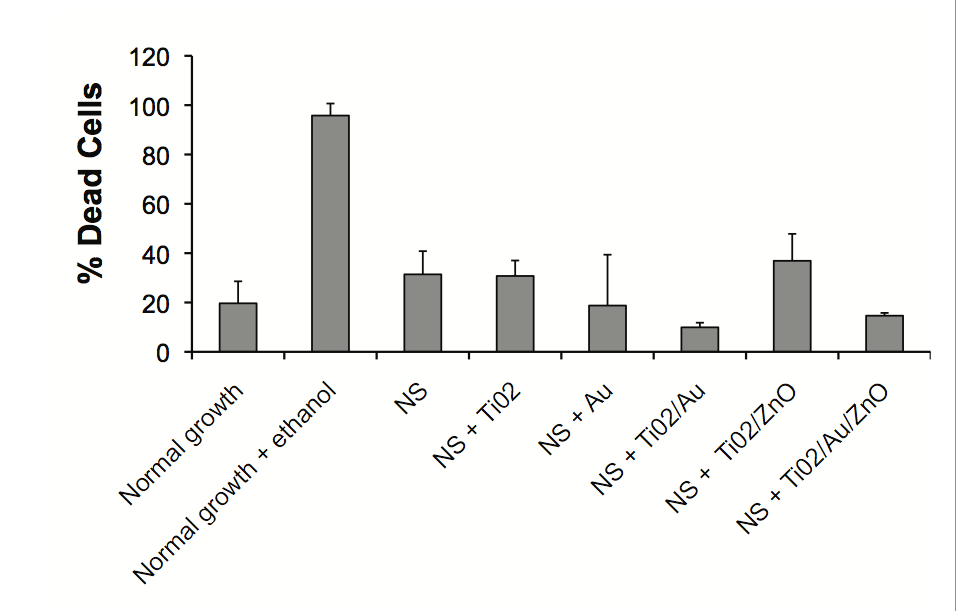

Supplement: Additional file 2 — Viability of osteoblasts grown on nanosprings. Cells were allowed to grow for 5 days on either plain glass (normal growth), or cover slips coated with gold nanoparticles (Au), titania (TiO2), titania and gold (TiO2/Au), titania and zinc oxide (TiO2/ZnO) or titania, gold particles and zinc oxide (TiO2/Au/ZnO). Cultures were stained with Vybrant Green (green), which labels the nuclei of all cells and with propidium iodide (red), which preferentially stains dead cells. The percentage of cells dead cells was determined for a total of 500 cells by dividing the number of propidium iodide positive cells by the number of Vybrant Green positive cells. Data were compiled from 3 independent experiments. Error bars represent standard deviation. As a positive control, we treated cells grown on normal growth conditions with ethanol, which induces death. [file 1477-3155-10-6-S2.DOC]

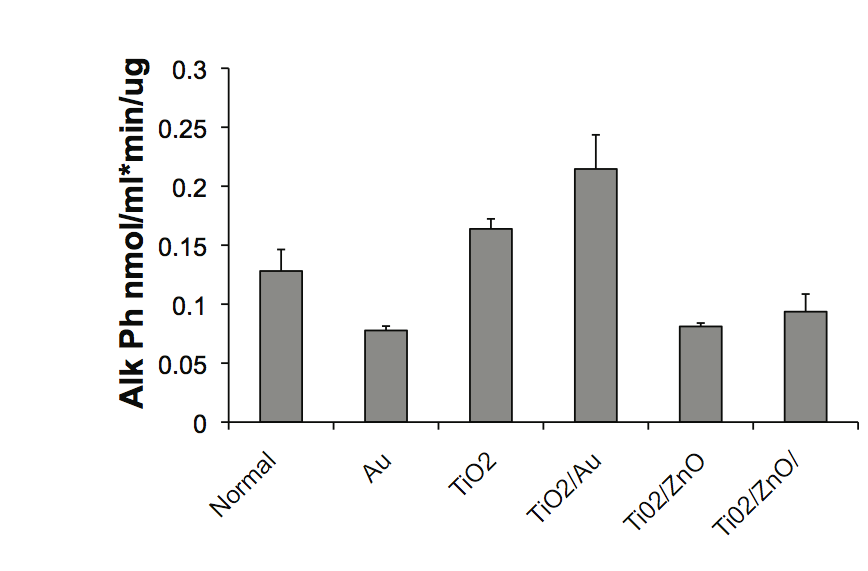

Supplement: Additional file 3 — Effect of metal coatings on osteoblast differentiation. Cells were grown for 36 days under normal growth conditions or glass cover slips coated with the metals indicated in the graph. Levels of alkaline phosphatase activity normalized to total amount of protein were calculated for each sample. Bars represent average of 3 independent experiments. Error bars are the standard deviation. [file 1477-3155-10-6-S3.DOC]
